# Evaluation of right ventricular performance and impact of continuous positive airway pressure therapy in patients with obstructive sleep apnea living at high altitude

**DOI:** 10.1038/s41598-020-71584-9

**Published:** 2020-11-19

**Authors:** Ai-Ai Chu, Hong-Mei Yu, Hui Yang, Li-Min Tian, Zhong-Yuan Hu, Na Jiang, Wan-Xia Xie, Yan Huang

**Affiliations:** 1 Department of Cardiology, Gansu Provincial Hospital, No. 204 Donggang West Road, Chengguan District, Lanzhou, 730000 China; 2 Department of Neurological Rehabilitation, Gansu Provincial Hospital, Lanzhou, China; 3 Department of Cardiology, Guangzhou Red Cross Hospital, Jinan University, Guangzhou, Guangdong China; 4 Department of Endocrinology, Gansu Provincial Hospital, Lanzhou, China; 5 Department of Anesthesiology, Gansu Provincial Hospital, Lanzhou, China; 6 Heart Center, The First Hospital of Lanzhou University, Lanzhou University, Lanzhou, China

**Keywords:** Cardiology, Medical research

## Abstract

Obstructive sleep apnea syndrome (OSAS) can lead to alterations in right ventricular (RV) performance and pulmonary vascular haemodynamics. Additionally, altitude-related hypoxia is associated with pulmonary vasoconstriction, and the effect of high-altitude on the pulmonary circulation in OSAS patients can be further altered. We sought to assess alterations in RV morphology and function in OSAS patients living at high altitude by way of 2-dimensional speckle tracking echocardiography (2D-STE), real-time 3- dimensional echocardiography (RT-3DE) and cardiac biomarkers. We also evaluate the impact of continuous positive airway pressure (CPAP) treatment on RV performance. Seventy-one patients with newly diagnosed OSAS and thirty-one controls were included in this study. All individuals were assessed for cardiac biomarkers as well as underwent 2D-STE and RT-3DE. Forty-five OSAS patients underwent CPAP therapy for at least 24 weeks and were studied before and after CPAP treatment. RT-3DE was used to measure RV volume, and calculate RV 3D ejection fraction (3D RVEF). Peak systolic strain was determined. Cardiac biomarkers, including C-reactive protein (CRP), N-terminal pro-B-type natriuretic peptide, and cardiac troponin T were also measured. Right atrium volume index, RV volume, RV volume index, systolic pulmonary artery pressure (sPAP), pulmonary vascular resistance (PVR) and level of serum CRP were significantly higher in OSAS group, while OSAS patients showed lower 3D RVEF and RV longitudinal strains. Compared to the patients with sPAP < 40 mmHg, RV longitudinal strains in patients with sPAP ≥ 40 mmHg were lower. Both RV global longitudinal strain and sPAP were associated with apnea–hypopnea index. Patients treated with 6 months of CPAP therapy had significant improvement in RV geometry and performance. RV structural abnormalities and RV function impairments were observed in OSAS patients living at moderate high altitude compared to control highlanders. The reversibility of these changes after application of CPAP were further confirmed.

## Introduction

Obstructive sleep apnea syndrome (OSAS), which is characterized by periodic upper airway obstruction during sleep, waking up repeatedly from sleep and excessive daytime sleepiness, is a common clinical form of sleep disordered breathing^1^. It is associated with an increased risk of cardiovascular morbidity and mortality^2^. OSAS is also independently associated with the occurence of cardiovascular disease such as atrial fibrillation^3^, coronary artery disease^4^, heart failure^5^ and stroke^6^.

Several observations have confirmed the adverse effects of OSAS on the left ventricle. For example, LV systolic function and diastolic function were both adversely affected, that was, global longitudinal strain (GLS) and LV ejection fraction decreased^7,8^. However, there are few reports indicating the effect of OSAS on the right ventricular morphology and function. The exact pathophysiological mechanism of right ventricle (RV) remodeling in OSAS patients is unclear, but studies have shown that elevated sPAP and hypoxic-oxidative stress caused by apnea/hypopnea may be related to it^9^.

Hypoxia is a strong stimulus to pulmonary artery vasoconstriction, which causes increased pulmonary artery pressure and pulmonary vascular resistance, and further leads to adaptive changes of RV^10^. Compared to healthy highlanders, even OSAS patients who lived at moderate high altitude had shown higher systolic pulmonary pressure and more significant RV remodeling^11^. Previous studies have assessed the impact of OSAS treatment with continuous positive airway pressure (CPAP) on the functional changes of the left ventricle^12,13^ and right ventricle^14,15^, confirming the improved LV and RV function. However, the degree of RV alterations in OSAS patients who lived at mid-high altitudes with CPAP treatment remains to be studied.

The right ventricle (RV) has a complex anatomical structure with a "crescent shape" in the cross-section, which contains a large number of muscle trabeculae and thick control cords. In addition, the ventricle is located behind the sternum. It is difficult to assess RV geometry and function with conventional echocardiography. Two-dimensional speckle tracking echocardiography (2D-STE)^16^ and real-time 3-dimensional echocardiography (RT-3DE)^17^ are independent on the assumption of cardiac geometry and have no angular dependence. Therefore, in theory, both 2D-STE and RT-3DE are suitable for evaluating the structure and function of right ventricle.

The right ventricular volume and ejection fraction measured by RT-3DE have a good correlation with the "gold standard" results measured by cardiac magnetic resonance (cMRI)^18^. In addition, serum C-reactive protein (CRP), N-terminal pro-B-type natriuretic peptide (NT-proBNP), and cardiac troponin T (cTnT) levels were previously evaluated in OSAS patients^19–22^. Although cardiac biomarkers are useful in the diagnosis of many cardiovascular disease, their roles in evaluating OSAS-associated cardiac dysfunction remain controversial^23–26^, not to mention in high altitude settings.

We hypothesized that the use of new echocardiographic techniques and cardiac biomarkers could assess changes in RV geometry and function in patients with OSAS living at moderate high altitude, and that RV structural abnormalities and dysfunction could also be reversed by CPAP treatment.

## Methods

### Study population

The present study was conducted in accordance with the principles of Declaration of Helsinki. Gansu provincial people’s hospital ethics committee approved the protocol, and written informed consent was obtained from all enrolled subjects.

A cohort of newly diagnosed patients confirmed by polysomnography were recruited and referred to Sleep Center of Gansu provincial people's hospital, China. Healthy subjects were from physical examination Center of Gansu provincial people’s hospital. Exclusion criteria were: chronic lung disorders, congestive heart failure, cardiomyopathies, previous myocardial infarction, more than mild valvular heart disease and atrial fibrillation. All OSAS patients meet the following criteria: (i) all selected candidates were born in Lanzhou (Lanzhou, between 1,500 and 3,000 m), and (ii) all selected candidates should not live at a lower or higher altitude for a long time (6 months or more)^11^.

### Polysomnography

All enrolled subjects (including healthy subjects) underwent polysomnographic recording using ALICE5 (Hanfei, Shanghai) for a full-night within 24 h of admission. Obstructive apnea was defined as: compared with the baseline, after at least 10 s of breathing effort, the peak deviation of the oral and nasal heat sensor decreased b  ≥ 90%, at least 50% of the airflow decreased, and at least 3% of the oxyhemoglobin saturation decreased. The number of apnea/hypoventilations per hour was defined as apnea–hypopnea index (AHI).OSAS was defined as ≥ 5 AHI events per hour in the presence of clinical symptoms suggesting OSAS^27,28^. If the patient's AHI is greater than or equal to 5, it can be diagnosed as OSAS. Patients with AHI values between 5 and 15 are considered mild, patients with AHI values between 15 and 30 are considered moderate, and patients with AHI values greater than or equal to 30 are considered severe OSAS^29^. Forty-five compliant patients with OSAS who had performed CPAP (Philips DS500) therapy and completed RT-3DE and 2D-STE analyses were studied before and after treatment. Patients who received CPAP treatment were instructed to use the CPAP device nightly for at least 6 months, and echocardiograms were repeated on the second day after the last nocturnal CPAP treatment while patients were awake.

### Echocardiography

Echocardiographic data were obtained using the EPIQ 7C (Kininklijke Philips NV, Eindhoven, The Netherlands) within 24 h of admission. The mean values of all echocardiography data were measured for three consecutive cycles. The examination was carried out by two experienced cardiologists who were unware of the existence of the disease or the severity of the individual.

RV diameters (including RV apicobasal diameter, RV mediolateral diameter and RV long-axis diameter) were measured in apical 4-chamber view. RV thickness was measured in sub-costal view when the tricuspid leaflet was parallel to the free wall of RV. Right atrium volume (RAV) was calculated by the area-length method in apical 4-chamber view and the right atrial volume indices (RAVI) were derived by dividing the RAV by the body surface area (BSA). RV global systolic function was assessed by measuring M-mode derived tricuspid annular systolic excursion (TAPSE). In apical 4-chamber view, early diastolic (E) and atrial (A) peak velocities (m/s) and E/A ratio at the tips of the tricuspid leaflets were recorded by pulsed Doppler. Pulsed Tissue Doppler of the lateral tricuspid annular was to measure systolic (S’), early diastolic (e’) and atrial (a’) velocities (all in cm/s). The E/e’ ratio was obtained by dividing E by e’. The sum of the Bernoulli equation ([tricuspid valve velocity, jet velocity]^2^ × 4) and the average pressure of the right atrium yields sPAP. Estimate the right atrial pressure by the quantitative assessment of inferior vena cava (3 or 8 or 15 mm Hg in relation with size and inspiration at rest and during forced inhalation)^30^. Place the pulse wave Doppler at the proximal end of the RV outflow tract on the short axis side of sternum to obtain the RV outflow tract time–velocity integral (RVOT-VTI). The pulmonary vascular resistance was determined using the equation: pulmonary vascular resistance = peak tricuspid regurgitant velocity/RVOT-VTI × 10 + 0.16^31^.

**Figure 1:**
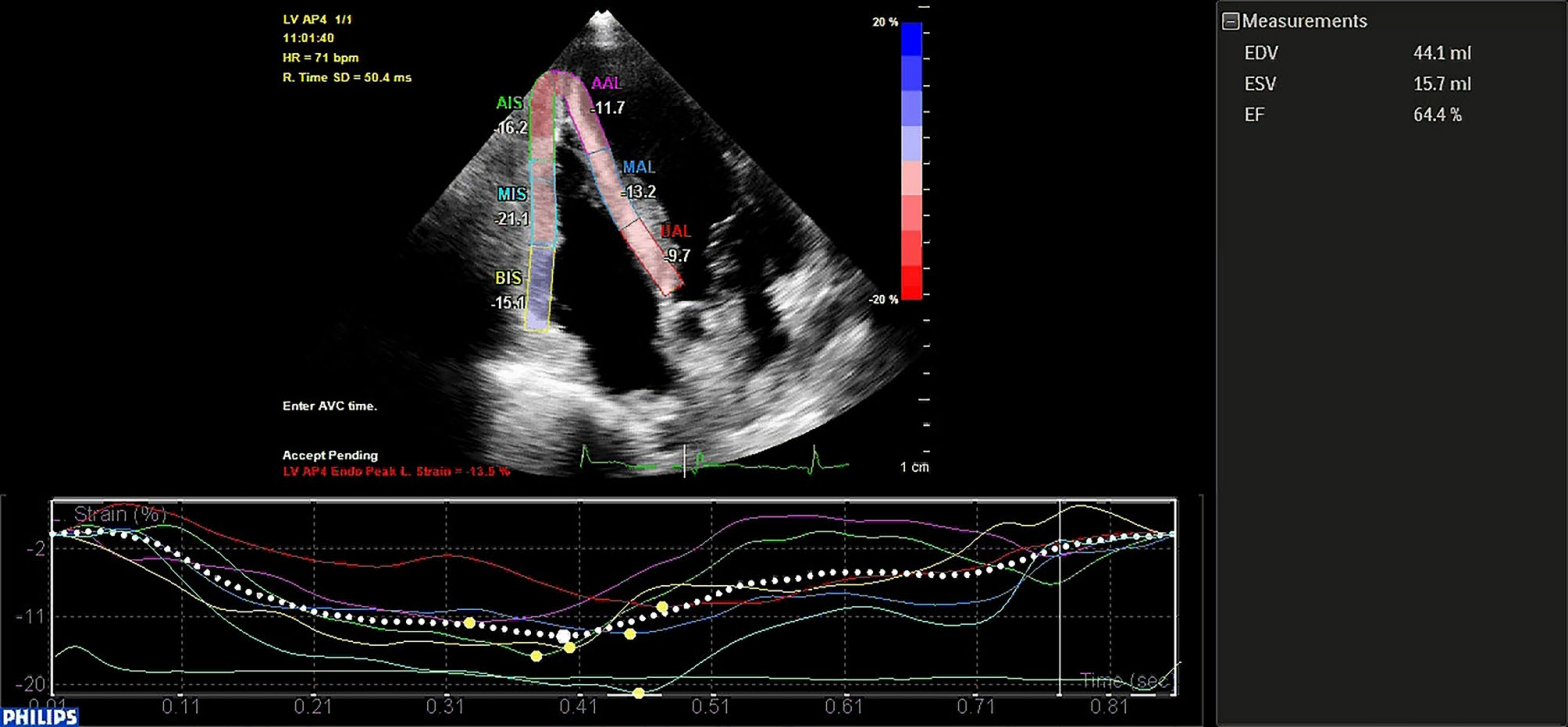
Speckle-tracking echocardiography analysis in an OSAS patient: the right ventricle is divided in 6 segments: 3 on the free wall and 3 in the septum.

Peak systolic longitudinal strain of the RV free wall (Fig. 1) was measured in the 4-chamber view using speckle-tracking analysis^32^. 2D-STE was performed using the commercially available software QLAB Advanced Tissue Motion Quantification (Philips) equipped with STE analysis. The software analyzes motion by tracking the motion of natural acoustic markers between frames in a two-dimensional space. At the end of the systole, manually track the right ventricle rim and adjust the automatically generated region of interest according to the thickness of the myocardium. RV global longitudinal strain (RV GLS) was the average of 6 segments of the RV lateral wall and interventricular septum. Free lateral longitudinal strain (RV LLS) was the average of 3 segments of lateral wall and septal longitudinal strain (RV SLS) was the average of 3 segments of interventricular septum^39^.

**Figure 2:**
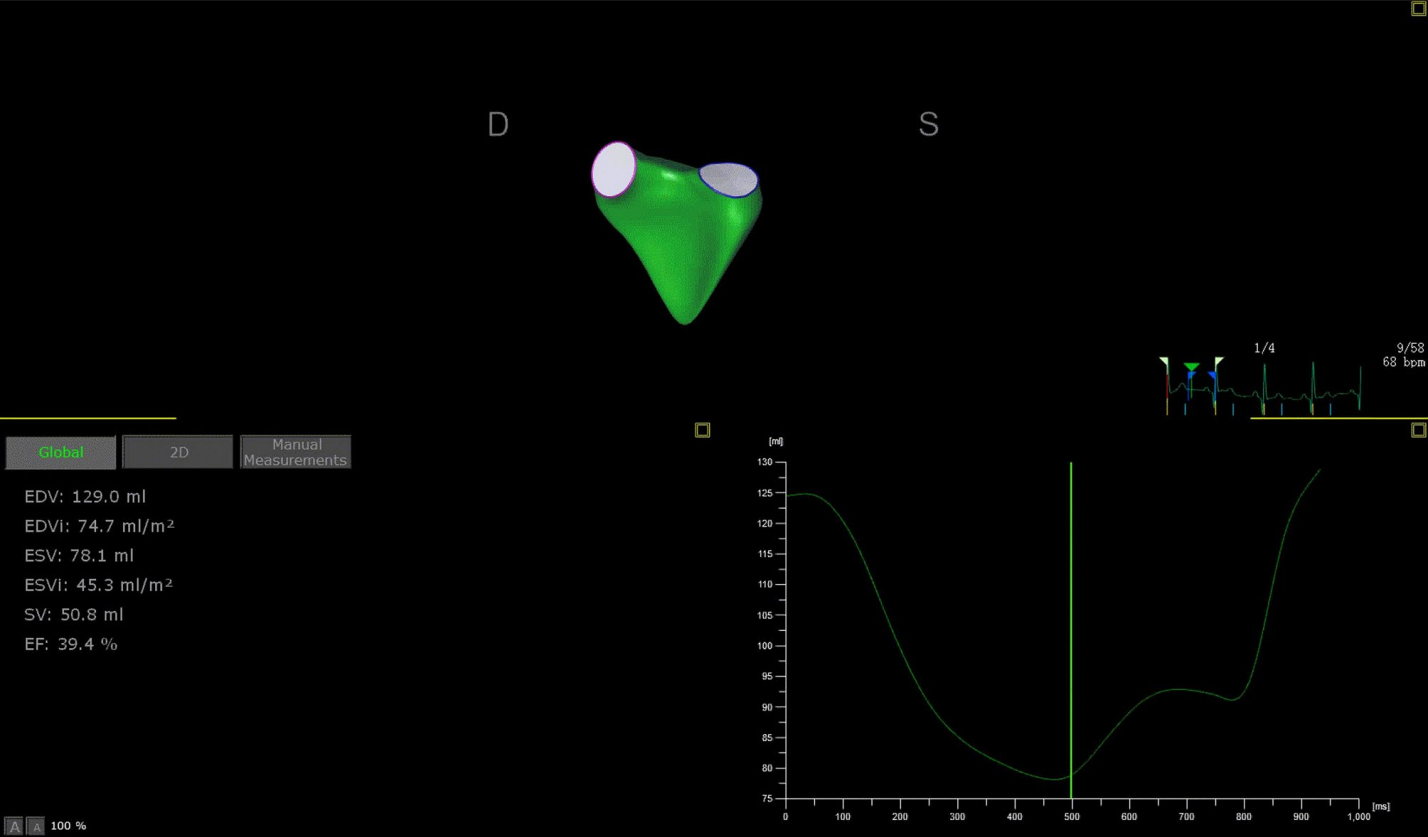
An example of OSAS patient for calculation of right ventricular end-diastolic and end-systolic volumes utilizing three-dimensional echocardiography.

RV 3D images (Fig. 2) were obtained by a volumetric 3D transducer (1–3 MHz) from apical four-chamber view. Individuals were instructed to hold their breath, and images were coupled with electrocardiographic recordings. All images are recorded with at a frame rate of at least 30 frames for reliable analysis. Semiautomatic endocardial border tracking and RV volume calculations were performed by 4D RV quantification software (Tomtec Imaging Systems, Gmbn, Unterschleissheim, Germany), which has been validated by cardiac MRI^18,33^. The RV end-diastolic volumes (RVEDV) and end-systolic volumes (RVESV) were determined by visual inspection throughout the cardiac cycle. The RV end-diastolic volumes indexs (RVEDVI) and RV end-systolic volumes indexs (RVESVI) were derived by dividing the RVEDV by the BSA and by dividing the RVESV by the BSA, respectively.

### Cardiac biomarkers analysis

Plasma and serum samples were stored for a maximum of 2 weeks at − 20 °C before being moved to a − 80 °C freezer. Serum levels of CRP were measured with latex agglutination immunoassay (Mitsubishi Kagaku Yatoron; Tokyo, Japan). NT-proBNP levels were measured with an electrochemiluminescence sandwich immunoassay (Elecsys ProBNP; Roche Diagnostics Inc). Quantitative determination of cTnT levels was performed using a third-generation Roche Elecsys assay.

### Statistical analysis

All statistical analysis was performed with SPSS 19.0 for Windows software in a compatible computer. Data for continuous variables are presented as the mean ± SD or median and interquartile range, and categorical variables are presented as frequencies and percentages. One-way Kolmogorov–Smirnov test is conducted to test normal distribution within the groups. For the parameters of the normal distribution, the independent-samples *t* test is used for comparison, and for the parameters of the non-normal distribution, the Mann–Whitney *U* test is used for comparison. For comparisons involving categorical variables, use the chi-square test or Fisher's exact test based on the expected cell count. Linear regression analysis was used to evaluate the correlation between AHI and clinical variables, and the Pearson (*r*) coefficient values were obtained. Independent links between variables were determined by multiple linear regression analysis.. The coefficient of variation was used to assess the intra- and interobserver variabilities for RV GLS, RV LLS, RVEDV, RVESV and RVEF assessed by 2D-STE and RT-3DE in a sample of 20 patients.

All statistical tests were two-sided, and a *p* value < 0.05 was considered statistically significant.

## Results

A total of 80 patients with first OSAS were initially evaluated. Nine patients were excluded: 5 (8%) patients did not have sufficient image quality for tracking of RV walls. Another 4 (7%) patients refused to enroll in the study. Thus, 71 patients were enrolled in the present study. Mean age was 48.0 ± 7.8 years, and 52 patients were males. Thirty-one patients matched to age and sex were control group.

Detailed demographic, clinical information and polysomnographic data from both OSAS patients and controls are displayed in Table 1. There were no significant differences in demographic/clinical variables such as age, gender, smoking, diabetes, hypertension, triglyceride, cholesterol, low-density lipoprotein ( LDL) and high-density lipoprotein (HDL) between the two groups, but body mass index (BMI) was significantly higher in OSAS patients. Compared with the control group, AHI was significantly increased and the minimal saturation of arterial oxygen (S_a_O_2_) and time S_a_O_2_ < 90% significantly decreased in the OSAS group.

**Table 1:** Clinical and demographic characteristics in control and OSAS groups.

	Control group (n = 31)	OSAS group (n = 71)	*P* value
Age (years)	47.2 ± 8.6	48.0 ± 7.8	0.67
Male gender (%)	19 (61.3%)	52 (73.2%)	0.87
Body mass index (kg/m^2^)	24.9 ± 2.3	26.0 ± 2.4	0.03
Body surface area (m^2^)	1.8 ± 0.1	1.8 ± 0.2	0.09
Smoker	11 (35%)	28 (39%)	0.86
Hypertension	8 (26%)	21 (30%)	0.98
Diabetes mellitus	4 (13%)	10 (14%)	0.89
Heart rate (beat/min)	69 ± 7	71 ± 6	0.11
Cholesterol (mg/dl)	159.8 ± 9.7	162.2 ± 6.6	0.19
LDL cholesterol (mg/dl)	79.9 ± 6.7	80.6 ± 6.1	0.65
HDL cholesterol (mg/dl)	39.6 ± 3.5	38.9 ± 2.9	0.34
Triglyceride (mg/dl)	116.8 ± 9.1	120.6 ± 9.0	0.07
AHI (events/hour)	2.6 ± 1.0	44.3 ± 17.2	< 0.001
Awake S_a_O_2_ (%)	96.4 ± 2.2	96.4 ± 2.1	0.99
Minimal S_a_O_2_ (%)	85.8 ± 3.8	75.3 ± 8.2	< 0.001
Time S_a_O_2_ < 90% (mins)	0.3 ± 0.3	22.7 ± 12.5	< 0.001
CRP (mg/L)	2.0 ± 0.5	5.4 ± 1.1	< 0.001
NT-proBNP (pg/ml)	794.2 ± 165.8	815.1 ± 115.3	0.51
cTnT (ng/ml)	< 0.01	< 0.01	1.00

*LDL* low-density lipoprotein, *HDL* high-density lipoprotein, *AHI* apnea–hypopnea index, *S*_*a*_*O*_*2*_ saturation of arterial oxygen, *CRP* C-reactive protein, *NT-proBNP* N-terminal pro-B-type natriuretic peptide, *cTnT* cardiac troponin T.

The baseline RV echocardiographic parameters are summarized in Table 2. No significant differences were present for the three diameters of RV (basal, middle and longitudinal) between the two groups, but right atrium volume index (RVAI) significantly increased in OSAS group compared with the control group. Systolic pulmonary arterial pressure (sPAP) and pulmonary vascular resistance (PVR) of OSAS patients was significantly higher than that of the control group. High sPAP were defined as sPAP ≥ 40 mmHg and were present in 30% of the control group and 48% of patients within the OSAS group. There was a significant difference in the proportion of high sPAP between the two groups. However, no differences were observed including TAPSE, E/A ratio, S_a_ velocity, E/e’ value and RVOT-VTI. OSAS patients had lower RV GLS and RV LLS but not RV SLS than controls. These differences in RVEF, RVEDV, RVESV, RVEDVI and RVESVI were also observed in OSAS patients (Table 2).

**Table 2:** RV echocardiographic variables in control and OSAS groups.

	Control group (n = 31)	OSAS group (n = 71)	*P* value
RAVI (ml/m^2^)	29.3 ± 4.7	37.7 ± 4.4	< 0.001
LVEF (%)	63.5.3 ± 4.9	63.0 ± 4.6	0.67
RV apicobasal diameter (mm)	35.1 ± 4.0	36.3 ± 4.2	0.21
RV mediolateral diameter (mm)	30.0 ± 3.9	30.5 ± 3.3	0.55
RV long-axis diameter (mm)	67.2 ± 5.0	68.2 ± 4.4	0.38
RVWT (mm)	4.0 ± 0.7	4.4 ± 0.6	0.01
TAPSE (mm)	20.7 ± 2.3	20.0 ± 2.2	0.18
Outlet RV VTI (cm)	13.2 ± 2.5	13.5 ± 2.5	0.49
TV E/A	1.2 ± 0.3	1.2 ± 0.2	0.89
TV S_a_ (cm/s)	13.2 ± 2.5	13.6 ± 2.4	0.49
TV E/e’	3.9 ± 0.7	4.3 ± 1.0	0.12
Systolic PAP (mmHg)	29.6 ± 4.7	42.7 ± 8.4	< 0.001
High sPAP	9 (30%)	34 (48%)	0.001
PVR (Woods)	1.7 ± 0.3	1.9 ± 0.3	< 0.001
RV GLS (%)	23.1 ± 3.8	18.8 ± 5.9	0.002
RV LLS (%)	25.7 ± 2.9	22.8 ± 3.4	< 0.001
RV SLS (%)	18.6 ± 5.2	18.8 ± 4.4	0.86
RVEDV (ml)	82.2 ± 11.1	99.7 ± 13.3	< 0.001
RVESV (ml)	41.2 ± 6.7	58.5 ± 11.1	< 0.001
RVEDVI (ml/m^2^)	45.9 ± 6.7	45.9 ± 6.7	< 0.001
RVESVI (ml/m^2^)	23.0 ± 4.0	31.8 ± 6.2	< 0.001
RVEF (%)	49.4 ± 3.4	41.5 ± 4.8	< 0.001

*RAVI* right atrial volume index, *LVEF* left ventricular ejection fraction, *RV* right wall, *RVWT* right ventricular wall thickness, *TAPSE* tricuspid annular plane systolic excursion, *E/A* ratio of early diastolic flow to atrial flow, *S*_*a*_ annular systolic velocity, *E/E*_*a*_ ratio of early diastolic flow to annular diastolic velocity, *PAP* pulmonary artery pressure, *PVR* pulmonary vascular resistance, *GLS* global longitudinal strain, *LLS* lateral longitudinal strain, *SLS* septal longitudinal strain, *RVEDV* right ventricular end-diastolic volume, *RVESV* right ventricular end-systolic volume, *RVEDVI* right ventricular end-diastolic volume index, *RVESVI* right ventricular end-systolic volume index, *RVEF* right ventricular ejection fraction.

By subdividing OSAS patients according to sPAP, 34 patients with sPAP ≥ 40 mmHg showed lower RV GLS and RV LLS than 37 patients with sPAP < 40 mmHg. However, there were no significant difference in RVEF, RVEDV and RVESV between the two groups.

Although there were no significant differences in NT-proBNP and cTnT between the two groups, serum CRP was significantly higher in the OSAS group than in the control group (Table 1). At 6 months after CPAP treatment, CRP levels were significantly lower than baseline (Table 3).

**Table 3:** Changes in RV parameters, cardiac biomarkers and polysomnographic data before and after CPAP therapy.

	Before CPAP therapy (n = 45)	After CPAP therapy (n = 45)	*P* value
RAVI (ml/m^2^)	37.2 ± 4.9	32.9 ± 3.9	< 0.001
RV apicobasal diameter (mm)	34.4 ± 3.4	35.3 ± 4.3	0.37
RV mediolateral diameter (mm)	30.8 ± 3.4	29.7 ± 2.9	0.14
RV long-axis diameter (mm)	67.9 ± 4.6	66.8 ± 3.9	0.28
RVWT (mm)	4.3 ± 0.5	4.2 ± 0.5	0.29
TAPSE (mm)	19.9 ± 2.1	19.8 ± 2.4	0.95
RV VTI (cm)	13.5 ± 2.4	13.9 ± 2.0	0.46
TV E/A	1.3 ± 0.2	1.2 ± 0.2	0.95
TV S_a_ (cm/s)	15.1 ± 2.7	14.5 ± 2.0	0.41
TV E/e’	5.4 ± 1.5	4.9 ± 1.0	0.54
Systolic PAP (mmHg)	44.6 ± 8.0	37.9 ± 6.9	0.001
High sPAP	22 (49%)	14 (31%)	0.001
PVR (Woods)	2.0 ± 0.4	1.7 ± 0.3	< 0.001
RV GLS (− %)	20.3 ± 3.3	23.1 ± 3.1	0.001
RV LLS (− %)	20.7 ± 3.5	23.0 ± 3.0	0.006
RV SLS (− %)	18.3 ± 4.2	18.8 ± 4.1	0.63
RVEDV (ml)	101.7 ± 13.2	92.6 ± 11.9	0.005
RVESV (ml)	57.3 ± 10.4	47.0 ± 9.4	< 0.001
RVEDVI (ml/m^2^)	55.5 ± 7.0	50.6 ± 7.0	0.007
RVESVI (ml/m^2^)	31.2 ± 5.6	25.7 ± 5.4	< 0.001
RVEF (%)	43.9 ± 5.2	49.5 ± 4.9	< 0.001
AHI (events/hour)	49.7 ± 16.4	4.9 ± 1.7	< 0.001
Awake S_a_O_2_ (%)	93.0 ± 1.9	93.6 ± 1.8	0.27
Minimal S_a_O_2_ (%)	72.5 ± 8.4	84.3 ± 5.8	< 0.001
Time S_a_O_2_ < 90% (min)	24.3 ± 12.0	3.4 ± 2.0	< 0.001
CRP (mg/L)	5.7 ± 0.8	2.4 ± 1.1	< 0.001
NT-proBNP (pg/ml)	554.5 ± 101.3	633.3 ± 115.4	0.24
cTnT (ng/ml)	< 0.01	< 0.01	1.00

*RAVI* right atrial volume index, *RV* right wall, *RVWT* right ventricular wall thickness, *TAPSE* tricuspid annular plane systolic excursion, *E/A* ratio of early diastolic flow to atrial flow, *S*_*a*_ annular systolic velocity, *E/E*_*a*_ ratio of early diastolic flow to annular diastolic velocity, *PAP* pulmonary artery pressure, *PVR* pulmonary vascular resistance, *GLS* global longitudinal strain, *LLS* lateral longitudinal strain, *SLS* septal longitudinal strain, *RVEDV* right ventricular end-diastolic volume, *RVESV* right ventricular end-systolic volume, *RVEDVI* right ventricular end-diastolic volume index, *RVESVI* right ventricular end-systolic volume index, *RVEF* right ventricular ejection fraction, *AHI* apnea–hypopnea index, *S*_*a*_*O*_*2*_ saturation of arterial oxygen, *CRP* C-reactive protein, *NT-proBNP* N-terminal pro-B-type natriuretic peptide, *cTnT* cardiac troponin T.

The effects of > 6 months CPAP therapy on the echocardiographic and polysomnographic variables were listed in Table 3. We observed the following changes after CPAP therapy: (A) decrease in sPAP, the proportion of high sPAP and pulmonary vascular resistance; (B) reduction in RVAI, RVEDV, RVESV, RVEDVI and RVESVI; (C) increase in RV GLS, RV LLS and RVEF; (D) decrease in AHI and time S_a_O_2_ < 90%; (E) increase in minimal S_a_O_2_. However, we did not observe any significant changes in clinical data before and after CPAP treatment, including BMI, body surface area (BSA), heart rate, cholesterol, LDL cholesterol, HDL cholesterol and triglyceride.

On correlation analysis, the following echocardiographic variables correlated with AHI: RVEF (*r* = −0.66, *p* = 0.03), RV GLS (*r* = −0.74, *p* = 0.02), RV LLS (*r* = −0.55, *p* = 0.04), sPAP (*r* = −0.67, *p* = 0.01) and RVOT-VTI (*r* = −0.36, *p* = 0.04). In forward stepwise multivariate linear regression analysis, RV GLS (*β* = −0.61, *p* = 0.01), RV LLS (*β* = −0.65, *p* = 0.01) and sPAP (*β* = 0.63, *p* = 0.001) were independently related to AHI with a regression model *r* = 0.56.

The intraobserver coefficients of variation for RV GLS, RV LLS, RVEDV, RVESV and RVEF were 3.1%, 4.2%, 4.3%, 3.5% and 5.2%, respectively. The interobserver variability was also low, with coefficients of variation of 4.2%, 4.9%, 3.5%, 5.6% and 6.0% when assessing RV GLS, RV LLS, RVEDV, RVESV and RVEF.

## Discussion

The main finding of the present study, by combining 2D-STE and RT-3DE, are as follows: (1) A significant reduction in RV function (including RV GLS, RV LLS and RVEF) and increase in RV volume (both RVEDVI and RVESVI) were observed in OSAS patients compared with healthy controls; (2) RV GLS and RV LLS were significantly lower in patients with sPAP ≥ 40 mmHg compared to those with sPAP < 40 mmHg; (3) In the absence of significant changes in NT-proBNP and cTnT, serum CRP was significantly higher in the OSAS group than in the control group; (4) both RV GLS and sPAP were independently associated with AHI; (5) CPAP treatment could improve RV structure and function in OSAS patients living at high altitude.

SomeOSAS patients (17–52%) exhibit pulmonary artery hypertension (PAH) during daytime^34–36^. It is well known that pulmonary artery pressure could increase during apneas, but the mechanism of daytime PAH in patients with OSAS is still under discussion^37–39^. In the present study, we demonstrated that sPAP (42.7 ± 8.4 mmHg) and pulmonary vascular resistance (PVR, 1.9 ± 0.3 Woods) are significantly higher in the OSAS patients than in the control highlanders. The most likely primary cause of the OSAS-related increase in PVR and sPAP is hypoxemia. We recognize that pulmonary vasoconstriction is a response of the body to acute hypoxia, and it can also regulate capillary perfusion to adapt to alveolar ventilation^40^. Meanwhile, Experimental studies of simulated repeated nocturnal apnea in mice have shown that intermittent hypoxia may induce pulmonary vasoconstriction, which in turn leads to pulmonary hypertension and increases right ventricular afterload^41,42^. Previous studies had confirmed the effect of obese or OSAS patients living in high altitude areas on pulmonary artery pressure^11,37^. This study found that an sPAP ≥ 40 mmHg was present in 30% of the healthy group and in 48% of the OSAS patients, which were lower than the data for OSAS patients living at high altitudes^11^ and were markedly higher than the data for the healthy population^37^. In summary, the degree of pulmonary vasoconstriction is closely related to hypoxemia, thereby increasing the pulmonary artery pressure. Recently, Guvenc et al. demonstrated that compared to healthy highlanders, the significant increase in pulmonary artery pressure (38.35 ± 8.60 mmHg) was observed in OSAS patients, whose pulmonary artery pressure seemed to be slightly lower than our results (42.7 ± 8.4 mmHg)^11^. In the present study, we displayed that sPAP (42.7 ± 8.4 vs. 31.9 ± 7.6 mmHg, *p* < 0.001) and pulmonary vascular resistance (PVR, 1.9 ± 0.3 vs. 1.7 ± 0.3 Woods, *p* < 0.001) are significantly higher in the OSAS patients than in the healthy group. Therefore, our results not only support the view of adverse effects of hypoxemia, but also show the fact that sPAP is markedly higher in OSAS patients than in healthy group.

Both STE and RT-3DE have been used to evaluate RV function in many cardiovascular diseases, including OSAS and atrial septal defect (ASD)^43–45^. Previous study pointed out that 3D-RVEF and apical strain were the more sensitive predictors of unfavorable outcome after atrial septal defect closure compared to 2D-Doppler indexes^43^. Recently, Buonauro and colleagues demonstrated reductions of RV GLS and RV LLS in 59 OSAS patients compared to healthy controls, and found that RV GLS was independently related to the level of sPAP and the severity of AHI. However, there was no difference in 3D-RVEF between the two groups^45^. In our present study, we not only confirm the reduction of RV GLS and RV LLS between the two groups, but also display that 3D-RVEF in OSAS patients have a significant reduction. It is well known that 3D-RVEF has a stronger correlation with MRI compared with traditional echocardiographic indicators, such as right ventricular fractional area change (RVFAC), the TAPSE and myocardial performance index (MPI)^43^. Interestedly, 34 patients with sPAP ≥ 40 mmHg do not show significant changes of 3D RVEF whereas RV GLS and RV LLS were were markedly lower than 37 patients with sPAP < 40 mmHg, which is agreement with previous study^45^.

Previous studies had demonstrated changes in right ventricular structure and function in patients with OSAS^11, 47^. Oliviera et al. had revealed that 56 patients with OSAS had significantly increased RV end-diastolic and end-systolic volumes and volumes indexes by using 3-DE^47^. More recently, Guvenc et al. had found that RV end-diastolic and end-systolic volumes indexes were significantly higher in OSAS patients than healthy highlanders^11^. In addition, Li et al. used velocity vector imaging (VVI) technology to evaluate the changes in the RA volume and function of OSAS patients^46^. Our study also finds that the RA and RV volumes and volumes indexes have significantly increased in OSAS patients. In our opinion, the most reasonable explanation for RA and RV dilatation is the increase in sPAP during the daytime in OSAS patients. However, previous studies on OSAS patients with normal daytime PAP had also revealed RV remodeling and dysfunction^47–49^, which provided evidence that other factors could have adverse effects.

Furthermore, in multiple linear regression analysis, we found that RV GLS and sPAP are independently associated with AHI. Overall, the results of our study suggested that nocturnal apneas have a potentially harmful effect on the RV. The cause can be partly explained by that RV afterload is increased due to the elevation of sPAP, and then leading to geometrical and functional changes of RV^41,42,50^. In addition, during sleep, patients with OSAS experience periodic upper airway obstructions that cause a sudden decrease in intrathoracic pressure, which increases venous blood return and RV volume^51^. At last, the recurrent PAH can also induce a decrease in coronary artery blood flow in the RV, and then affect myocardial contractility of the RV^52^.

In the present study, we find that CRP levels are significantly higher in OSAS patients than in control group, whereas NT-proBNP and cTnT levels did not differ significantly between the two groups, which is agreement with the findings by Roche et al.^53^ and Jane et al.^50^. It is believed that repeated hypoxic stress and sleep deprivation may be a pathophysiological mechanism leading to elevated CRP levels in patients with OSAS. Repeated hypoxic stress can not only lead to increased sympathetic nerve activity and endothelial dysfunction, but also induce the release of cytokines and the activation of inflammatory cells^26^. This may be the cause of increased morbidity and mortality in of atherosclerosis and cardiovascular diseases in OSAS patients^26^. Akashiba et al. found that CPAP treatment did not reduce CRP levels in OSAS patients^54^. Becker et al. showed that the levels of CRP and interleukin (IL)-6 in patients with OSAS could be decreased by CPAP treatment in agreement with our results but (IL)-6 not be measured in our study^55^. Effective CPAP treatment can improve the sleep quality of OSAS patients and reduce the decrease of nocturnal oxygen saturation, thereby reducing the body's hypoxic stress, reducing sympathetic nerve excitement, and further reducing the release of CRP.

Researches that CPAP can improve LV and RV function indicate the most direct evidence that OSAS causes cardiovascular abnormalities^12,56^. Magalang et al. reported that the end-diastolic volume of RV decreased significantly in OSAS patients who received CPAP treatment for 3 months^57^. In our study, after > 6 months treatment with CPAP, RV morphology and function had significantly improved, including decreased RA and RV volumes, increased RV ejection fraction and improved RV strains, which indicated a physiopathologic association between RV performance and OSAS. The CPAP may reduce excessive negative intrathoracic pressure, leading to a reduction in apnea-hypoxic events associated with hypoxemia, subsequently reducing pulmonary vascular resistance^58^. The lower RV afterload would allow a smaller RV volume and lager RV ejection fraction. In addition, Romero-Corralm and colleagues report that the treatment effect of CPAP on patients with OSAS is similar to that of chronic beta-bolckers on heart failure patients^14^. In the present study, we found a significant change in RV structure and performance after CPAP treatment. However, we did not compare the treated CPAP patients with those without CPAP after 24 weeks to verify the effects of CPAP. Although all of our subjects were OSAS patients living at high altitude, there was also no comparison between CPAP at high altitudes and CPAP at sea level to verify the effects of high altitude on pulmonary vessels and RV.

Some limitations should be discussed. First, this is a single-center experience, with a small number of individuals enrolled. Second, the potential limitation is that there are some clinical factors in the population, such as hypertension, BMI and aging.. Therefore, selection bias and potential selection bias should be taken into account when interpreting our findings. Additionally, one limitation was the difficult to obtain quality images from the selected population.

## Conclusions

We demonstrate that OSAS patients living at high altitude have increased sPAP, PVR and 3D RV volume, as well as RV dysfunction, which suggests that they respond to overload by OSAS disease. Furthermore, sPAP increase and RV GLS impairment are both associated with the severity of OSAS (as expressed by AHI and its grading). The detection of CRP levels may enable us to determine whether OSAS is associated with cardiovascular diseases, and CPAP treatment significantly reduces CRP levels, possibly further reducing the incidence of cardiovascular disease. Long-term CPAP treatment can reverse RV structural and functional abnormalities, and may improve clinical outcomes.
